# A Tank Bromeliad Favors Spider Presence in a Neotropical Inundated Forest

**DOI:** 10.1371/journal.pone.0114592

**Published:** 2014-12-10

**Authors:** Yann Hénaut, Bruno Corbara, Laurent Pélozuelo, Frédéric Azémar, Régis Céréghino, Bruno Herault, Alain Dejean

**Affiliations:** 1 El Colegio de la Frontera Sur, Departamento de Conservación de la Biodiversidad, Quintana Roo, Chetumal, Mexico; 2 Université Blaise Pascal, Laboratoire Microorganismes, Génome et Environnement, Clermont-Ferrand, France; 3 Centre National de la Recherche Scientifique, Unité Mixte de Recherche 6023, Université Blaise Pascal, Aubière, France; 4 Université Paul Sabatier, Laboratoire Écologie Fonctionnelle et Environnement, Toulouse, France; 5 Centre National de la Recherche Scientifique, Unité Mixte de Recherche 5245, Laboratoire Ecologie Fonctionnelle et Environnement, Toulouse, France; 6 Centre de coopération internationale en recherche agronomique pour le développement, Unité Mixte de Recherche 93, Ecologie des Forêts de Guyane, Campus Agronomique, Kourou, France; 7 Ecologie des Forêts de Guyane, Campus agronomique, Kourou, France; University of Minnesota, United States of America

## Abstract

Tank bromeliads are good models for understanding how climate change may affect biotic associations. We studied the relationships between spiders, the epiphytic tank bromeliad, *Aechmea bracteata*, and its associated ants in an inundated forest in Quintana Roo, Mexico, during a drought period while, exceptionally, this forest was dry and then during the flooding that followed. We compared spider abundance and diversity between ‘*Aechmea*-areas’ and ‘control-areas’ of the same surface area. We recorded six spider families: the Dipluridae, Ctenidae, Salticidae, Araneidae, Tetragnathidae and Linyphiidae among which the funnel-web tarantula, *Ischnothele caudata*, the only Dipluridae noted, was the most abundant. During the drought period, the spiders were more numerous in the *Aechmea*-areas than in the control-areas, but they were not obligatorily associated with the *Aechmea*. During the subsequent flooding, the spiders were concentrated in the *A. bracteata* patches, particularly those sheltering an ant colony. Also, a kind of specificity existed between certain spider taxa and ant species, but varied between the drought period and subsequent flooding. We conclude that climatic events modulate the relationship between *A. bracteata* patches and their associated fauna. Tank bromeliads, previously considered only for their ecological importance in supplying food and water during drought, may also be considered refuges for spiders during flooding. More generally, tank bromeliads have an important role in preserving non-specialized fauna in inundated forests.

## Introduction

Confronted with global warming, species may survive if their climate envelopes are wide enough to buffer new environmental conditions or if they are capable of moving to more suitable areas. For instance, in high latitude regions, sedentary animal species respond by the slow, poleward shift of their ranges [1], [2]. In tropical areas, animal species are mostly affected by major changes in precipitation related to El Niño and La Niña episodes [3] and can eventually adapt to their new conditions by moving to a different microhabitat (physiological adaptations and microevolution, if any, occur over much larger time scales). Since 1976, there has been an increase in the frequency and intensity of El Niño and La Niña episodes related to global warming [4]. These episodes correspond to sea-surface temperature anomalies across the tropical Pacific Ocean which impact the atmospheric circulation worldwide, causing environmental changes with opposing effects [5]. In the Yucatan Peninsula, this phenomenon will be amplified in the future because this area is one of the most responsive tropical regions to changes in global climate; there, decreases in precipitation during La Niña episodes result in severe droughts [6].

In this context of climate change and intensification of drought events, inundated forests constitute relevant systems for understanding how species can adapt to recurrent drought by modifying their utilization of the various habitats found in this environment. More specifically, we studied spider distribution in an inundated forest in southern Quintana Roo, Mexico in May 2011 after a La Niña episode while, exceptionally, the ground was dry, and then in January 2012 when the forest was again flooded.

The inundated forests of Quintana Roo are characterized by an abundance of orchid and bromeliad epiphytes, including the large tank bromeliad, *Aechmea bracteata*. Indeed, tank bromeliads provide habitat, food and water to numerous aquatic and terrestrial organisms (e.g., microbes, algae and various animals such as spiders, crustaceans, insects, mollusks, amphibians, reptiles and mammals) [7]–[11]. Furthermore, they are interesting models for studying biotic interactions because they form spatially discrete and highly replicated micro-ecosystems [12]. For instance, *A. bracteata* provides ants with a nesting place and, in turn, obtains protection from defoliators and likely nutriments from the ants’ refuse (myrmecotrophy) [7]. On the other hand, spiders, including the Ctenidae, Theraphosidae and Salticidae, can be associated with bromeliads because the plant’s humid micro-climate helps them to avoid desiccation, while providing an adequate reproduction site and a good foraging area with abundant prey [13]–[17]. Also, certain spider species select specific rosette and leaf characteristics [18]–[20]. Note that spiders and certain ant species are generalist predators competing for the same resources and can also prey on one another [21]. Indeed, ants have frequently been observed preying on spiders, including web-weaving spiders [21]–[23]. Whereas the majority of web-weaving spiders discard the ants that fall onto their web, some *Nephila* learn how to manipulate and capture the ants [24], [25]. Among the non-web-weaving spiders, some species from the families Ctenidae and Thomisidae occasionally prey on ants [26], whereas specialization in ant predation has been noted in the Zodariidae and Salticidae [27]–[29].

We specifically addressed the following questions. (1) Do *A. bracteata* bromeliads play a role in spider distribution in a context of a marked dry/wet succession in an inundated forest? (2) Do ants associated with *A. bracteata* impact spider presence?

## Materials and Methods

### Ethics Statement

This study was conducted according to relevant national and international guidelines. Permit #FAUT-0241 granted to Dr Yann Hénaut, issued by the *Secretaría de Medio Ambiente y Recursos Naturales* (SEMARNAT), according to the *Norma Oficial Mexicana* (NOM-126-ECOL-2000).

### Study site

This study was conducted in an inundated forest dominated by 10-m-tall *Metopium brownei* (Anacardiaceae) situated in southern Quintana Roo, Mexico (1.5 km from the Mayan ruins at Kohunlich; 54 km from Chetumal; 18.426725° N; 88.804360° W; 120 meters a.s.l). The mean temperature varies from 25.5 to 26.5°C. The dry season lasts from February to May and the rainy season from June to January. Hurricanes commonly occur in this area between May and December, and particularly in September.

### The plant

*Aechmea bracteata* is a large tank bromeliad (leaves *ca*. 1-m-long; inflorescences up to 1.7-m-long) found from Mexico to northern Colombia, from sea level to 1000 m. a.s.l [30]. Each plant is constituted of a group of shoots at different stages of maturity that develop from a rhizome [31]. As the rhizome grows, each shoot goes through the same stages, from the formation of the bud, which gives rise to a new shoot, up to the development of the inflorescence. Each shoot forms a rosette with numerous reservoirs (phytotelmata) where an abundant aquatic fauna develops. In the heart of the rosette, around the inflorescence, a central watertight cavity delimited by an amphora-shaped leaf is very often occupied by large ant colonies, mostly *Neoponera villosa* (Ponerinae) (until recently known as *Pachycondyla villosa* [32]) and *Dolichoderus bispinosus* (Dolichoderinae). When the ramet dries out and begins to rot, it becomes a refuge for ground-dwelling animals. Each plant therefore constitutes a complex ecosystem with its associated aquatic and terrestrial fauna [7].

### Data

**Figure 1:**
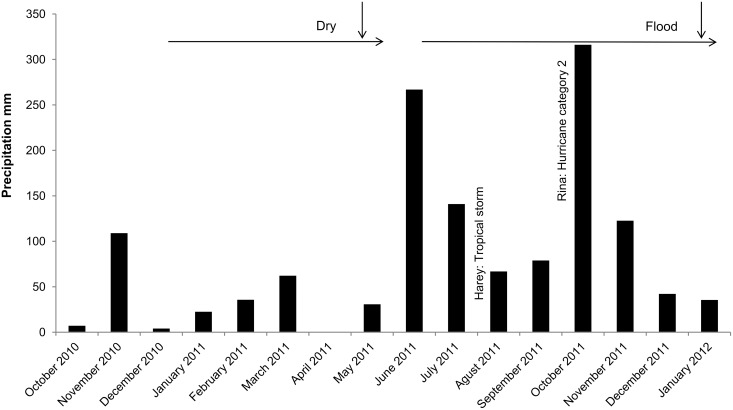
Precipitation registered in Chetumal by the Mexican national meteorological service. The vertical arrows indicate the collection dates. The horizontal arrows indicate the periods of drought and flooding. The name and category of climatic events are added alongside the corresponding bars.

We monitored 49 *A. bracteata* in May 2011 at the end of a pronounced drought where the forest floor had been dry for *ca*. 8 months and in January 2012 after heavy rains flooded the forest floor with 10–40 cm of water (see Fig. 1). Unfortunately, in the interim, heavy winds had caused some of the trees bearing *A. bracteata* to fall, explaining why the values are lower than 49 in January 2012.

In May 2011, while monitoring the 49 *A. bracteata*, we counted the number of shoots interconnected by a rhizome indicative of an individual (see [7]), measured their size (height and width), the height at which they were located on their host tree and noted what ant species they sheltered. Furthermore, we delimited a circle with a radius of 2 m around the base of each tree bearing an *A. bracteata*. To these “*Aechmea*-areas” corresponded “control-areas” of the same surface area situated ∼10 m further with, at their centers, a similar tree (same trunk diameter and inclination) but not bearing an *A. bracteata*. Due to the homogeneity of this forest, the two kinds of areas present the same ecological characters and are under the same climatic conditions.

During both the dry and the flooded periods, we carefully inspected all the *Aechmea*- and control-areas (on the ground and on the trees situated at the centers of these areas in May 2011 and on the trees only in January 2012) to look for spider presence. In the *Aechmea*-areas, we also noted if the spiders were in contact or not with the *A. bracteata* (i.e., crawling on them or their webs connected to the plant foliage).

The spiders were identified to the genus or species levels whenever possible. Yet, because individuals of the orb-weaving spiders were juveniles, their identification was based on their web architecture (i.e., vertical orb-web for the Araneidae, horizontal orb-webs for the Tetragnathidae and sheet-webs for the Linyphiidae). In May 2011, we did not collect the spiders so as not to disturb their community, whereas we collected voucher specimens for identification in January 2012.

### Statistical comparisons

We were basically interested in the differences in the number of spiders between *Aechmea*- and control-areas during the dry and the flooded periods. Because our experimental design uses only count variables with discrete, positive values, we modeled them with a Poisson distribution, and because the same *A. bracteata* were sampled twice (dry and flooded periods), we used the sampling units as a random factor in a generalized linear mixed-effects model. We firstly tested the effects of flooding and *Aechmea* and then the effects of flooding and contact with *Aechmea* (fixed effects in both cases) on the number of spiders (R v. 2.14.2 software; R Development Core Team [33]).

The Wilcoxon Matched Pairs test was used to compare spiders that live in direct contact with the *A. bracteata* foliage *vs*. orb-weavers and the impact of ants on spider presence.

To bring out the relationships between spiders, ants and the number of shoots for both sampling periods, we used the Self-Organizing Map algorithm (SOM, neural network [34]) presented in Appendix S1.

## Results

### Impact of *Aechmea bracteata* presence and flooding on spider distribution

**Figure 2:**
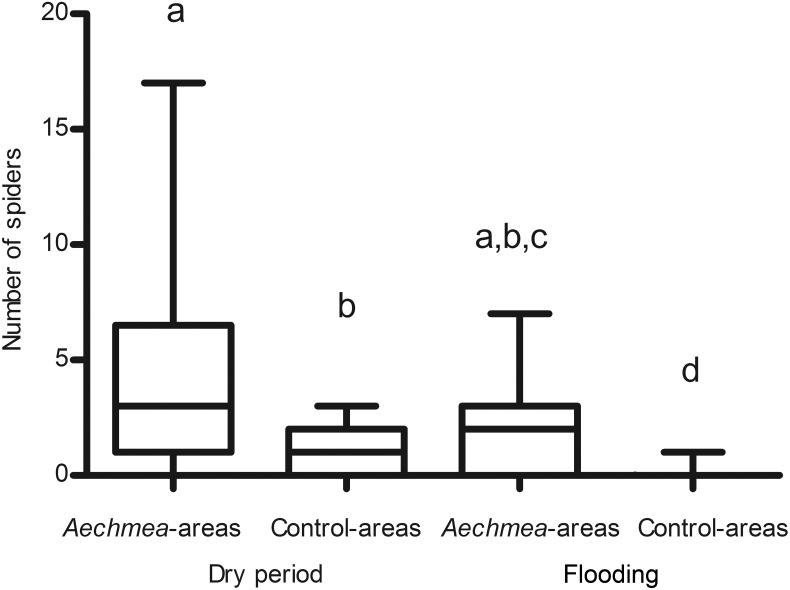
Effects of flooding and *Aechmea bracteata* on the number of spiders (modeled in a linear mixed-effects statistical framework where the individual samples were set as a random variable). Points refer to the observed numbers, vertical lines to the model prediction.

*Aechmea* presence has a significant positive effect on the number of spiders, the contrary being true for flooding (Table 1A), and *Aechmea-*areas harbored significantly more spiders than expected by chance during flooding (i.e., the interaction term was significantly positive in Table 1A; see also Fig. 2).This situation was mostly due to a decrease in *Ischnothele caudata* (Dipluridae), the only funnel-web tarantula noted, which represented 70% of the spiders recorded during the dry period and 42% during flooding. The Ctenidae (banana spiders) were represented by *Cupiennius salei*, the Salticidae by the genera *Thiodina*, *Lysomanes* and *Menemerus*, the Tetragnathidae by the genus *Leucauge*, while the Araneidae and Linyphiidae were not identifiable at the genus level.

**Table 1:** Effects of flooding and *Aechmea bracteata* presence (A) and of flooding and contact with *A. bracteata* foliage (B) on the number of spiders (modeled in a linear mixed-effects statistical framework where the individual samples were set as a random variable).

	Variable	Estimate	Z value	*P*
A	Intercept	0.036	0.224	ns
	Flooding	−2.282	−4.706	***
	*Aechmea*	1.329	6.873	***
	Flooding*Aechmea	1.518	3.023	**
B	Intercept	0.238	1.423	ns
	Flooding	−1.455	−5.851	***
	Contact	0.358	1.577	ns
	Flooding*Contact	1.142	3.982	***

### Direct association with *Aechmea bracteata* in the context of a drought and flooding

**Figure 3:**
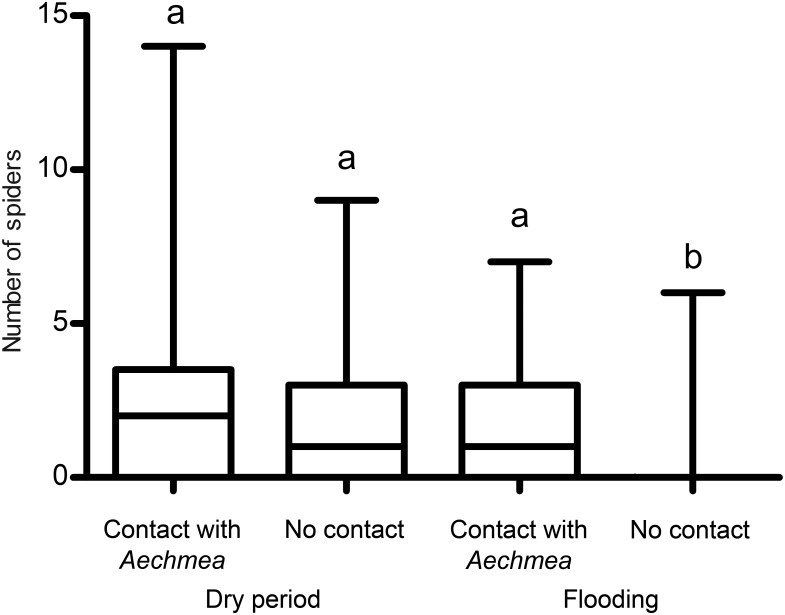
Effects of flooding and contact with *Aechmea bracteata* on the number of spiders (modeled in a linear mixed-effects statistical framework where the individual samples were set as a random variable). Points refer to the observed numbers, vertical lines to the model prediction.

Flooding has a significantly negative effect on the number of spiders, but we did not find an effect for contact with *A. bracteata per se* (Table 1B). However, the presence of *A. bracteata* significantly increased the number of spiders during flooding (spiders in direct contact with the plant), but not during the dry period (i.e., the interaction term was significantly positive in Table 1B; see also Fig. 3). Indeed, during flooding, *A. bracteata* individuals sheltered spiders regardless of whether they were orb-weaving or not (1.11±0.21 *vs*. 1.16±0.22; N = 37; Z = 0.02; P = 0.98). In contrast, during the drought, most of the spiders noted in direct contact with the *A. bracteata* foliage belonged to the Dipluridae, Ctenidae and Salticidae as opposed to the Araneidae, Tetragnathidae and Linyphiidae (i.e., orb-weavers) (1.49±0.3 *vs*. 0.30±0.08; N = 47; Wilcoxon Matched Pairs test: Z = 3.4; P<0.001).

### Spider presence in relationship to ant presence

Between the two sampling periods, the same *A. bracteata* individuals sheltered the same ant species: *Azteca* sp. (n = 5) and *Dolichoderus bispinosus* (n = 12) (both Dolichoderinae), and *Neoponera villosa* (Ponerinae) (n = 10). In 12 cases, two of these species shared the same *A. bracteata*, occupying different shoots (*Azteca* + *Neoponera*: n = 2; *Azteca* + *Dolichoderus*: n = 2; *Dolichoderus* + *Neoponera*: n = 8), but we did not record an ant colony in the remaining 10 *A. bracteata.* Also, *Camponotus* sp. (Formicinae) and *Pseudomyrmex* sp. (Pseudomyrmecinae) were each associated once with *Azteca* sp. and *N. villosa*, respectively.

During the dry period, we did not note a significant difference in the number of spiders (all species pooled) per *A. bracteata* whether the ants were present or not (2.80±0.60; N = 35 *vs*. 1.60±0.56; N = 10; U = 145; P = 0.41), while during flooding we noted significantly more spiders in the presence of ants (2.41±0.35; N = 29 *vs*. 0.90±0.30; N = 10; U = 69.5; P<0.05).

**Figure 4:**
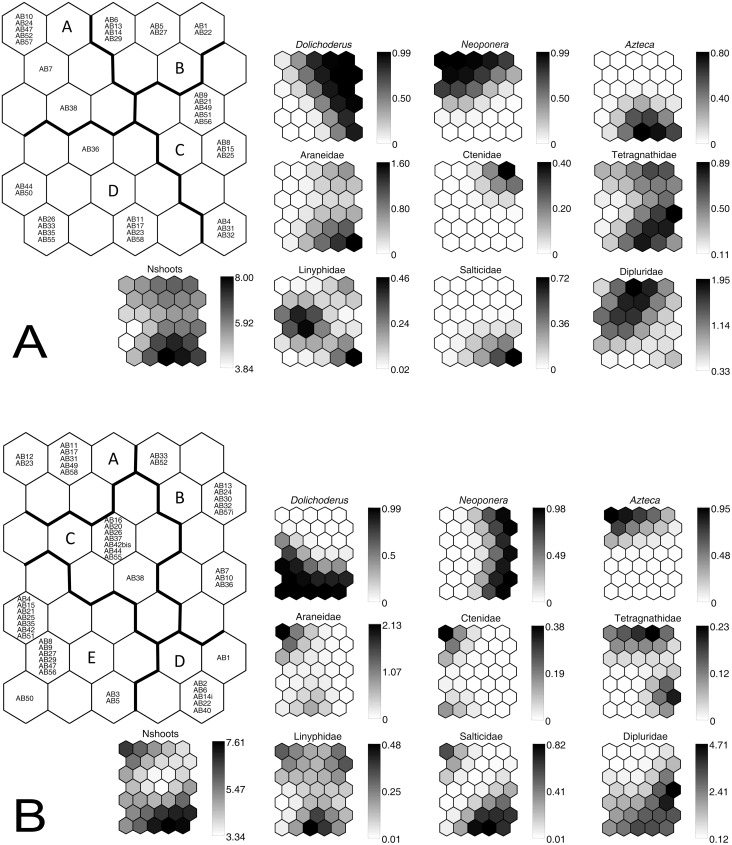
Using the Self-Organizing Map algorithm (SOM) to establish congruent patterns between spiders, ants and the bromeliad, *Aechmea bracteata*. Distribution of the *A. bracteata* individuals on the SOM during the dry (Fig. 4A, May 2011) and the flooding period (Fig. 4B, January 2012) according to their spider and ant assemblages. The numbers of *A. bracteata* shoots were given a null weight during the ordination process, and therefore act as an explanatory variable. In the large map, *A. bracteata* individuals that are neighbors within hexagons (or output neurons) are expected to have similar spider-ant assemblages, while those separated by a large distance from each other have different spider-ant assemblages. Clusters A–D (May 2011) and A–E (January 2012) were delineated by applying Ward’s algorithm to the weight vectors of the spider and ant species in the various hexagons. Each small map representing the number of shoots or one taxa can be compared to (or superimposed on) the corresponding large map representing the distribution of *A. bracteata*. They thus show gradients in the number of shoots (Nshoots), the probability of occurrence of each ant species (first line in the small maps), and the abundance of each spider taxa (second and third lines in the small maps) within the SOM (in shades of grey; dark  =  high values, light  =  low values). Codes (e.g., AB9) correspond to individual plants (sampling units).

We further illustrate the distribution patterns of ants and spiders among bromeliads using the SOM, knowing that the number of shoots has only a slight impact on spider presence (Fig. 4). Indeed, ant influence was highlighted for the Dipluridae whose presence was negatively associated with *Azteca* and positively associated with *Neoponera* and to a lesser degree with *Dolichoderus*, regardless of the season. The contrary was true for the Ctenidae in May, but not in January; however, the number of individuals recorded was very low (low values on the lateral shade scales of Fig. 4A). Among the web-weaving spiders, the Araneidae seem positively associated with *Azteca*, while the Tetragnathidae and Linyphidae were present regardless of the ant species occupying the *A. bracteata*. The Salticidae were positively associated with *Azteca* in January 2012 during flooding, but not in May 2011.

## Discussion

### Climatic conditions and spider abundance, distribution and relationship with *Aechmea bracteata*

During the dry season, *A. bracteata* permits spiders to survive a prolonged drought, something noted for other spider taxa [13]–[15]. Moreover, during flooding, the spiders, which can no longer live on the ground, also disappeared from trees devoid of an *A. bracteata*, even though these trees could have allowed them to escape the flooding. So, almost all of the spiders recorded were associated with *A. bracteata* which serves as a refuge.

The most abundant spider species noted in this study, the funnel-web tarantula, *I. caudata*, is a subsocial mygalomorph (Dipluridae) known for providing extensive maternal care and spinning thick curtain webs with a tubular retreat and a sticky sheet-like structure to catch prey [35]. Individuals of this species build a permanent web on the ground, under rocks, at the base of trees or can cover plants; in all cases, a cavity permits them to install their tubular retreat [35]. This was also the case in this study when the forest was dry, although *I. caudata* individuals were frequent in the *A. bracteata* foliage. However, during flooding, nearly only *A. bracteata* offers a suitable architecture for this spider species which can weave its tubular retreat in the base of the long and sturdy leaves.

The absence of spiders on trees other than those bearing *A. bracteata* during flooding could be the consequence of a ‘cleansing effect’ resulting from the heavy rains and strong winds that affected the spiders which were already installed on these trees as well as those which took refuge on them while the ground was flooded. It appears that the *A. bracteata* architecture offers a good shelter for spiders during these drastic periods (see also [36]). In addition, one must keep in mind that spider distribution is related to food location [37], so that spider association with *A. bracteata* is likely also related to prey abundance.

The majority of the spiders we observed, particularly the funnel-web tarantulas and the orb-weaving spiders, are known to live on supporting structures and in ecosystems different from those of the present study [23], [35]–[38]; in other words, they are not tank bromeliad specialists. On the contrary, the Ctenidae, including species of the genus *Cupiennius* (as noted in this study) and the Salticidae have already been reported as associated with bromeliads, but they may also live on other plants [13]–[16].

In the inundated forests along the Neotropical coasts affected by frequent climatic events such as hurricanes [6], large tank bromeliads, including *A. bracteata*, offer permanent shelter to a wide diversity of organisms. Indeed, they form islands for aquatic and terrestrial fauna, including arboreal ants [7], [9], [10], [39]. We show that, in contrast, *A. bracteata* acts as a refuge for spiders during flooding as in other climatic conditions they are not associated with bromeliads [13]–[16]; see also [40].

### Spider-ant relationships

It is known that, for spiders, ants represent the risk of being killed [22], [23], [41], although some spiders, such as *I. caudata*, frequently prey on ants [35]. The association of funnel-web tarantulas with particular ant species may be the result of predation by those spiders on the ants. Other spiders, such as the Ctenidae, are generalist predators that may prey on ants [26], whereas their great velocity, their ability to jump away [42] and their good vision [43], allow them to escape ant attacks and to co-exist with ants. Similar traits are known in the Salticidae, which are also good at avoiding ants thanks to their vision and ability to recognize ants [44]. Many orb-weavers do not prey on ants, carefully expulsing the ants that fall onto their web [24], [25]. Even when their webs are anchored on ant-occupied bromeliads, these spiders can share sites with ants thanks to the isolation provided by those webs [25]. In addition, the web silk of certain species repels ants [45].

Here we show that ant presence not only does not repel spiders but rather favors their presence and some specificity was even noted between certain ant and spider taxa. Thus, one can hypothesize that, at least for those species well adapted at avoiding ants, some spiders benefit from ant presence as protection from other enemies. Indeed, protection from predation through association with aggressive ants is exemplified when the orb-weaver, *Eustala oblonga* (Araneidae), inhabits an ant-acacia [46]. Also, *Phintella piatensis* (Salticidae) lives on the territory of the arboreal weaver ant, *Oecophylla smaragdina*, despite being occasionally preyed upon. In reality, it uses the ant scent as protection from its main predator, a spitting spider of the genus *Scytodes* [47]. Finally, myrmecomorphy, or ant-like appearance, permits some salticids to gain protection from other spiders through Batesian mimicry (a palatable spider avoids predation by resembling an unpalatable ant) [48], [49].

Therefore, *A. bracteata* individuals constitute ecological refuges during flooding, and their associated ants globally favor spider presence (see also [50]).

In conclusion, the nature of the relationship between spiders, *A. bracteata* and ants depends on climatic events and arthropod traits. *Aechmea bracteata* not only provides fauna with the habitat and water which allow them to avoid suffering from drought, but also has great importance in the preservation of biodiversity during flooding. Therefore, this tank bromeliad has an important role in preserving arthropod fauna in the inundated forests of Quintana Roo. This role should become more important in the coming years in a context of climate change as El Niño/La Niña events are expected to be more frequent and intense, which in this area will likely correspond to an increase in the intensity of droughts. Like many tank bromeliads, *A. bracteata* is well adapted to wet/dry extreme events and may mitigate the effects of climate change on the local arthropod fauna.

## Supporting Information

Appendix S1 **The Self-Organizing Map algorithm (SOM).** The SOM was used as an analytical tool to establish congruent patterns between spiders, ants, and variables characterizing the host plant, *Aechmea bracteata*.
(DOCX)
