# Supplementary material for: A Tank Bromeliad Favors Spider Presence in a Neotropical Inundated Forest
Source: PLoS One. 2014 Dec 10;9(12):e114592. doi: 10.1371/journal.pone.0114592 (PMC4262423; doi:10.1371/journal.pone.0114592)
Supplement: Appendix S1 — The Self-Organizing Map algorithm (SOM). The SOM was used as an analytical tool to establish congruent patterns between spiders, ants, and variables characterizing the host plant, Aechmea bracteata. (DOCX) [file pone.0114592.s001.docx]

**Appendix S1.** **The Self-Organizing Map algorithm (SOM)**. The SOM was used as an analytical tool to establish congruent patterns between spiders, ants, and variables characterizing the host plant, *Aechmea bracteata*.

To bring out the relationships between the spiders, ants, and the number of shoots of *Aechmea bracteata*, we used the Self-Organizing Map algorithm (SOM, neural network). Combining ordination and gradient analysis functions, the SOM is a convenient way to show patterns in high-dimensional ecological data in a readily interpretable manner, without prior transformation. The SOM algorithm is an unsupervised learning procedure that transforms multi-dimensional input data into a two-dimensional map subject to a topological (neighborhood preserving) constraint (details presented in [1]). The SOM thus plots the similarities of the data by grouping similar data items together onto a 2D-space (displayed as a grid) using an iterative learning process that was detailed in [2]. The SOM algorithm is especially relevant for analyzing sets of variables that vary and co-vary in a non-linear fashion, and/or that have skewed distributions (because of many zero values), and/or that belong to different categories (e.g., quantitative, semi-quantitative, qualitative). Additionally, the SOM algorithm averages the input dataset using weight vectors through the learning process and thus removes noise. A full description of the modeling procedure employed here (i.e., training, map size selection, number of iterations, map quality values) was provided in [3] and [4].

We aimed to illustrate the distribution patterns of ants and spiders as probability of occurrence (ants) and number of individuals (spiders) during two sampling periods (May 2011 and January 2012) taking into account the number of shoots per *Aechmea* as a potential explanatory variable. The structure of the SOM for each of these two analyses consisted of two layers of neurons connected by weights (or connection intensities): the input layer was composed of nine neurons (three ant and six spider species) connected to 37 (January) or 47 samples (May), and the output layer was composed of 35 or 30 neurons (May and January, respectively) displayed as hexagonal cells organized on a grid. The optimal number of output neurons for each period was chosen after testing quantization and topographical errors (see [2]). At the end of the training, each sample is set in a hexagon of the SOM map. Samples appearing distant in the modeling space (according to the variables used during the training) represent expected differences in the ant and spider assemblages for real environmental characteristics. Ward’s algorithm was applied to cluster the trained maps. The SOM units (hexagons) were divided into clusters according to the weight vectors of the neurons, and clusters were justified according to the lowest Davis Bouldin Index (i.e., for a solution with low variance within clusters and high variance between clusters [4]). To analyze the contribution of the various ants and spiders in clustering structures of the trained SOM, each input variable calculated during the training process was displayed in each neuron of the trained SOM in gray scale. This method clearly shows the discriminatory powers of input variables in mapping [1].

Second, we aimed to bring out the relationships between ant and spider distributions on the one hand, and the number of shoots on the other hand. During the above-mentioned training, we used a mask function to assign a null weight to the variable “number of shoots”; whereas the nine species were assigned a weight of ‘1’ so that the ordination process was based on these biological variables only. Setting the mask value to zero for a given component removes the effect of that component on the organization of the map [5], [6]. The values for the “number of shoots” were thus displayed on the SOM previously trained with ant-spider data only. This permitted a co-variation between these habitat and biological variables to be illustrated.

After training the SOM, the topographic error was null; the map thus reflects the typology of the input data very well, and so is relevant for subsequent interpretation (see [1]). Five and four clusters were delimited on the SOM both during the dry period in May 2011 and the flooding in January 2012, respectively; they clearly separated the ant species in May, but not so well in January (Figs. 4A and 4B).

**References**

[1] Kohonen T 2001. Self-Organizing Maps, 3^rd^ ed., Berlin; Springer. 501p.

[2] Céréghino R, Park YS, Compin A, Lek S (2003) Predicting the species richness of aquatic insects in streams using a limited number of environmental variables. J North Amer Benthol Soc 22: 442–456.

[3] Park YS, Céréghino R, Compin A, Lek S (2003) Applications of artificial neural networks for patterning and predicting aquatic insect species richness in running waters. Ecol Model 160: 265–280.

[4] Céréghino R, Park YS (2009) Review of the self-organizing map (SOM) approach in water resources: commentary. Environ Model Software 24: 945–947.

[5] Sirola M, Lampi G, Parviainen J (2004) Using self-organizing map in a computerized decision support system. In: Pal N, Kasabov N, Mudi R, Pal S, Parui S, editors. Neural information processing. Berlin: Springer-Verlag. pp. 136–141.

[6] Raivio K (2006) Analysis of soft handover measurements in 3G network. Proceedings of the 9th ACM international symposium on modeling analysis and simulation of wireless and mobile systems (Terromolinos, Spain, October 02-06, 2006). New York: ACM Press. pp. 330–337.
